# Impact of economic problems on depression in single mothers: A comparative study with married women

**DOI:** 10.1371/journal.pone.0203004

**Published:** 2018-08-24

**Authors:** Ga Eun Kim, Hee-Yeon Choi, Eui-Jung Kim

**Affiliations:** 1 Department of Psychiatry, College of Medicine, Ewha Womans University, Seoul, Korea; 2 Deadong Hospital, Daegu, Korea; Chiba Daigaku, JAPAN

## Abstract

**Objective:**

The purpose of this study was to investigate the risk factors that influence depression among single mothers.

**Methods:**

Participants were 195 single mothers and 357 married mothers living in an urban community in South Korea. All subjects completed self-report questionnaires that included the following self-rating scales: the Global Assessment of Recent Stress, the Center for Epidemiologic Studies-Depression scale, and the Korean version of the Alcohol Use Disorder Identification Test. Multiple logistic regression analysis was performed to examine independent factors affecting single mothers’ depression.

**Results:**

The prevalence of depression differed notably between the single mothers and the control group, at 33% and 8%, respectively. In the single mothers, young age, low income, residential instability, high stress, and high alcohol-related problems were determined to be associated with depression. Furthermore, after adjusting for covariates, living in rental housing (OR = 11.46, 95% CI 1.72–76.46) was found to be an independent risk factor of depression in the single mothers, while stress (OR = 1.16, 95% CI 1.09–1.24) was found to have this effect on the married mothers.

**Conclusions:**

Practical services concerning economic stability and parenting support should be provided for single mothers to reduce depression among this demographic.

## Introduction

The number of single-parent households in Korea has been rising in recent years, from 1,594,000 households in 2010 to 1,783,000 households in 2015. This trend is consequently increasing the share of single-parent households, which rose from 8.6% in 2005 to 9.2% in 2010, and further increased to 9.5% in 2015 [1].

According to the 2015 Single Parent Household Survey, which involved the participation of 2,552 single parents, the share of single-parent households created as a result of divorce was 77.1%, while the share of those created through spousal death was 15.8%, indicating that divorce is the predominant cause of single parenthood in Korea. The survey also found that, at 43.7%, the majority of single-parent households consisted of a mother and child [2]. Households headed by single mothers typically have reduced income, because in Korea fathers tend to be the primary economic providers. Furthermore, as the single parent family structure forces such parents to seek employment outside the home, single mothers are more likely to experience increased economic hardship and parenting stress [3], which also leads to an increased risk of employment and housing insecurity. Furthermore, for these women, the difficulty of finding appropriate childcare exacerbates the already challenging task of finding reliable and satisfactory employment. Of all single-parent households, those headed by mothers have the lowest monthly income. Moreover, 48.2% of single mothers who manage to find employment have been found to spend 10 hours or more at work every day and to receive insufficient holidays, which leaves a rather large void in childcare during which their children are alone without adult supervision. Compounding the economic and parenting difficulties hampering single mothers is weak social support, which increases the severity of various issues for both mothers and their children [2].

According to Crosier et al. [4], single-parent households headed by mothers are more likely to experience poor mental health than two-parent households; and the most significant contributors to such mental health issues are economic hardship and a lack of social support. Furthermore, Cairney et al. [5] reported that single-parent families are more likely to suffer from depression than two-parent families, and that single-parent families have an elevated level of chronic stress, experience weak social support, and have infrequent contact with friends and families. The 2015 Single Parent Household Survey found that 20.2% of single parents experience depressive symptoms, with 54% coping with the symptoms on their own and a mere 5.7% seeking professional help [2]. On the other hand, a study by Wang et al. [6] reported a contrasting finding to that of other previous research; according to their study, depressive symptoms are only significantly more prevalent in households headed by single mothers who are 25–50 years of age, and reported that economic hardship and weak social support are not correlated with depressive symptoms in these families.

Economic strain also has a significant effect on single parent families’ physical and mental well-being. In fact, 20.8% of single parents report feeling chronically unwell yet being unable to see a doctor, with 53.4% of these citing “economic strain” as the primary reason for not seeking medical advice, which is a higher percentage than the national average [2].

Although many studies have examined mental health in single-parent households, their findings regarding influencing variables are inconclusive. Single-parent families are socially disadvantaged and face various economic, psychological, and parenting difficulties, and to relieve these issues a multi-pronged social support system is urgently required. Identifying the variables influencing the mental health of single-parent families would be helpful for designing policies and services to promote mental health in such households. We wanted to identify the factors affecting depression in single mothers in Korea. Furthermore, identifying the factors influencing depression in single mothers compared to those in married mothers would be helpful for designing polices that can provide the practical support they really require.

Considering the above, this study aimed to examine single mothers, who account for the largest share of single parents, to identify the sociodemographic and psychological variables influencing depression in this population group. The identified variables were then compared to the variables that have been found to influence depression in married mothers of two-parent families to isolate those that are particular to single mothers

## Method

### Subjects

The current study’s survey was conducted from June 7, 2011, to June 24, 2011, as part of the “Community Survey Project for Health-care Plan Targeting Parents and Children of Single-parent Households” led by Yangcheon-gu mental health and welfare center. Yangcheon-gu is largely divided into three dongs (the smallest administrative unit): Mok-dong, Sinwol-dong, and Shinjeong-dong, and these three dongs are further divided into nineteen smaller dongs. The nineteen dongs were classified into these three district strata and stratified by age and residence type. A total of 497 single-parent households were selected by stratified random sampling. Thirty surveyors, who had received training in means of conducting home surveys, visited the households of the individual residents. After a brief introduction of the survey purpose and an explanation concerning the correct means of completing the questionnaire, written consent was collected and the self-report questionnaire was distributed. The completed questionnaires were collected on a subsequent visit. If the respondent was unavailable at the time of collection, a maximum of two more visits were made to collect the questionnaire. The response rate was 59%, reflecting the participation of a total of 291 single-parent households. Of these households, 195 single mother families were selected for the study, following the exclusion of single-parent families that were headed by unmarried mothers, single fathers, and grandparents.

For the control group, which comprised married mothers of two-parent families, a total of 1,000 community members were selected via stratified random sampling as part of the same city’s “2009 Mental Healthcare Project: Community survey”; they were then visited at their homes by the surveyors for 1:1 interviews. After excluding men and unmarried women, the data of 357 married women were used. The study was approved by Institutional Review Board of Ewha University Medical Center.

### Assessment method

The self-report questionnaire was designed to survey the respondents’ sociodemographic characteristics, including age, education level, occupation, average monthly household income, housing type, and religion, as well as other variables such as depressive symptoms, stress, and alcohol use. Housing types in Korea consist of ownership, Jeonse, monthly rental housing, and public rental housing. Jeonse is a system that involves a substantial deposit to the landlord, which is then returned when the contract is completed. Monthly rental housing is a system of paying monthly rent to the landlord. This monthly rent is not returned later. Public rental housing is a system in which national or private construction companies lease to low-income residents.

#### Center for epidemiologic studies-depression scale

To assess depressive symptoms, the Center for Epidemiologic Studies-Depression Scale (CES-D) was used, which is the most commonly used epidemiologic survey tool for all ages [7]. The CES-D is known to be particularly appropriate for use in epidemiological research because of its conciseness, and also because it assesses the severity of symptoms based on their duration. Although not a clinical diagnostic tool for depression, the CES-D effectively reflects the severity of depressive symptoms in community studies, and the tool’s reliability and validity have been proven by Cho and Kim [8]. The CES-D consists of 20 items, each of which is designed to be self-scored using a 0–3-point scale; thus, the total possible score is 0–60 points, with a higher score indicating a higher level of depressive symptoms. Sixteen points and 25 points are the most commonly used cut-off scores, signifying probable depression and definite depression, respectively. For the current study’s analysis, participants who received a CES-D score of 25 or above were classified into the depressed group, while those who scored less than 25 were assigned to the non-depressed group.

#### Global assessment of recent stress

The Global Assessment of Recent Stress (GARS), which was developed by Linn [9], consists of items assessing the level of stress perceived over the previous week. In the current study, the Korean-language version translated by Koh and Park was used [10]. This scale consists of a total of eight items, each of which is designed to be self-scored using a 0–9-point scale. The items consist of assessments of pressure related to work/job/school, interpersonal relationships, changes in relationships, sickness/injury, financial matters, unusual happenings, changes/no change in routine, and overall stress perception. The GARS does not have a cut-off point and higher scores indicate a greater level of perceived stress during the previous week.

#### Korean version of the alcohol use disorder identification test

The World Health Organization developed the Alcohol Use Disorder Identification Test (AUDIT) as a screening instrument for hazardous and harmful alcohol consumption [11]. The scale consists of a 10-item self-administered questionnaire designed rated on a scale from 0 to 4. The items consist of assessments of frequency of drinking, typical quantity, frequency of heavy drinking, impaired control over drinking, increased salience of drinking, morning drinking, guilt after drinking, blackouts, alcohol-related injuries, and other assessments related to drinking. The higher the total score, the greater the likelihood of hazardous and harmful alcohol use, as well as possible alcohol dependence [12]. We used the Korean version of this instrument by Lee et al. [13]. Korean respondents with a total score of 12 or higher are highly likely to have alcohol use disorders.

### Analysis

To examine whether there was a correlation between subjects’ sociodemographic characteristics and depressive symptoms, group differences in terms of continuous variables were tested using t-tests and one-way analyses of variance. Meanwhile, for group comparisons that included categorical variables, chi-square tests were performed. To examine the correlations between the continuous variables, Pearson correlation analysis was performed. Regarding the variables shown to be correlated with depressive symptoms, logistic regression analysis was conducted to determine those that exerted a significant influence on depressive symptoms (CES-D score of 25 points or higher). The variables found to be depression risk factors in the univariate logistic regression were then used as covariates in the multivariate logistic regression to identify the depression risk factors that were independently significant. All statistical analyses were performed using SPSS version 20.0 (IBM Corp., Armonk, NY), and statistical significance was set at p < 0.05.

## Results

### Comparison of sociodemographic characteristics and psychological factors between single and married mothers

The mean age of the single mothers (42.7 years) did not differ significantly from the mean age of the married mothers (43.3 years). Meanwhile, 67.2% of the single mothers had graduated from high school, and 26.2% from college. In the control group, the corresponding percentages were 53.2% and 36.1%, respectively, indicating a smaller share of individuals with higher educational attainment among the single mothers (p = 0.006). Regarding employment and occupation, while 66.1% of the mothers in the control group were homemakers, a mere 13.3% of the single mothers were. Furthermore, 63.6% of the working single mothers were employed in the service industry or held menial jobs. Over half of the households headed by single mothers had an average monthly income of less than two million KRW, and 36.4% had an average monthly income of two to five million KRW. On the other hand, 86.8% of the two-parent households had an average monthly income of two to five million KRW, whereas only 3.1% had an average monthly income of less than two million KRW, indicating a significantly higher proportion of households with a low average monthly income among the single mothers (p < 0.001). Finally, at 59%, the majority of households headed by single mothers lived in Jeonse rental housing (security deposit), while a mere 11.8% were home-owners; meanwhile, in the control group, 67.5% were homeowners while 29.1% lived in Jeonse rental housing. This indicates a low rate of home-ownership among the single mothers (p < 0.001) (Table 1).

**Table 1:** Sociodemographic characteristics and psychological variables of participants.

	Single mothers (N = 195) N (%)	Married mothers (N = 357) N (%)	p
*Sociodemographic characteristics*			
Age†	42.7 ± 7.8	43.4 ± 8.8	0.347
Education level			0.006*
Middle school graduate	13 (6.7)	38 (10.6)	
High school graduate	131 (67.2)	190 (53.2)	
Above college	52 (26.2)	129 (36.1)	
Occupation			< 0.001*
House wife, student	26 (13.3)	236 (66.1)	
Production	124 (63.6)	89 (24.9)	
Indoor job	40 (20.5)	25 (7.0)	
Profession	5 (2.6)	7 (2.0)	
Monthly Income (10 thousand won)			< 0.001*
< 200	113 (57.9)	11 (3.1)	
200–499	71 (36.4)	310 (86.8)	
≥ 500	11 (5.6)	36 (10.1)	
Residence			< 0.001*
Owner	23 (11.8)	241 (67.5)	
Jeonse rental housing	115 (59.0)	104 (29.1)	
Monthly rental housing	32 (16.4)	7 (2.0)	
Public rental housing	25 (12.8)	5 (1.4)	
Religion			0.410
No	83 (42.6)	147 (41.2)	
Yes	112 (57.4)	210 (58.8)	
*Psychological variables*			
CES-D score†	20.9 ± 8.3	12.5 ± 7.8	< 0.001*
GARS score†	33.1 ± 12.0	19.7 ± 10.4	< 0.001*
AUDIT-K score†	7.1 ± 7.1	5.8 ± 4.2	0.023*

*P < 0.05

† Mean ± SD (Standard deviation)

AUDIT-K: the Korean version of the alcohol use disorder identification test, CES-D: the center for epidemiologic studies depression scale, GARS: the global assessment of recent stress

The mean CES-D score for the single mothers was significantly higher than that for the married mothers in the control group (20.9 vs. 12.5, p < 0.001), and the mean GARS score for the single mothers was significantly higher than that for the married mothers in the control group (33.1 vs. 19.7, p < 0.001). Finally, the mean AUDIT-K score for the single mothers was also significantly higher than that for the married mothers in the control group (7.1 vs. 5.8, p = 0.023) (Table 1). The AUDIT-K cut-off score indicating alcohol use disorder in Korean women was 12 points. Among single mothers, 25.6% were estimated to have alcohol use disorder, which was significantly higher than 8.4% in the control group (p < 0.001).

### Comparison of the sociodemographic characteristics and psychological variables that contribute to depression among single and married mothers (Table 2)

To analyze the factors contributing to depression, the subjects were assigned to the depressed or non-depressed group based on the CES-D cut-off score for definite depression (25 points). Of a total of 195 single mothers, 65 had a CES-D score of 25 points or higher, resulting in a depression prevalence of 33%; on the other hand, only 29 of the 357 married mothers had a score exceeding the cut-off point, resulting in an 8% prevalence. This indicated a significantly higher prevalence of depression among the single mothers (p < 0.001).

**Table 2:** Sociodemographic characteristics and psychological variables between the depression and non-depression groups of single and married mothers.

	Single mothers (N = 195)			Married mothers (N = 357)		
	Depression (N = 65) N (%)	Non- depression (N = 130) N (%)	p	Depression (N = 29) N (%)	Non- depression (N = 328) N (%)	p
*Sociodemographic characteristics*						
Age†	40.3 ± 7.8	44.0 ± 7.5	0.002*	43.4 ± 8.9	43.4 ± 8.8	0.993
Education level			0.260			0.364
Middle school graduate	2 (3.1)	11 (8.5)		1 (3.4)	37 (11.3)	
High school graduate	43 (66.2)	88 (67.7)		18 (62.1)	172 (52.4)	
Above college	20 (30.8)	31 (23.8)		10 (34.5)	119 (36.3)	
Occupation			0.375			0.881
House wife, student	10 (15.4)	16 (12.3)		20 (69.0)	216 (65.9)	
Production	43 (66.2)	81 (62.3)		7 (24.1)	82 (25.0)	
Indoor job	12 (18.5)	28 (21.5)		2 (6.9)	23 (7.0)	
Profession	0	5 (3.8)		0	7 (2.1)	
Monthly Income (10 thousand won)			0.004*			0.404
< 200	47 (72.3)	66 (50.8)		2 (6.9)	9 (2.7)	
200–499	18 (27.7)	53 (40.8)		25 (86.2)	285 (86.9)	
≥ 500	0	11 (8.5)		2 (6.9)	34 (10.5)	
Residence			< 0.001*			0.570
Owner	2 (3.1)	21 (16.2)		18 (62.1)	223 (68.0)	
Jeonse rental housing	33 (50.8)	82 (63.1)		10 (34.5)	94 (28.7)	
Monthly rental housing	12 (18.5)	20 (15.4)		0	7 (2.1)	
Public rental housing	18 (27.7)	7 (5.4)		1 (3.4)	4 (1.2)	
Religion			0.069			0.157
No	33 (50.8)	30 (38.5)		15 (51.7)	132 (40.2)	
Yes	32 (49.2)	80 (61.5)		14 (48.3)	196 (59.8)	
*Psychological variables*						
CES-D score†	30.5 ± 3.9	16.1 ± 5.2	< 0.001*	31.3 ± 7.1	10.8 ± 5.2	< 0.001*
GARS score†	40.1 ± 11.3	29.5 ± 10.7	< 0.001*	36.3 ± 12.0	18.2 ± 8.9	< 0.001*
AUDIT-K score†	9.5 ± 8.4	6.0 ± 6.1	0.003*	8.0 ± 6.2	5.6 ± 4.0	0.183

^*^P < 0.05

† Mean ± SD (Standard deviation)

Depression: CES-D score ≥ 25, Non-depression: CES-D score < 25. AUDIT-K: the Korean version of the alcohol use disorder identification test, CES-D: the center for epidemiologic studies depression scale, GARS: the global assessment of recent stress.

The mean age of the depressed single mothers was 40.3 years, which was significantly younger than the 44 years for the non-depressed single mothers (p = 0.002). Among the married mothers in the control group, however, the depressed and the non-depressed shared a mean age of 43.4 years. For the single and married mothers alike, no significant differences in education level, employment, or religion were found between the depressed and the non-depressed.

While 72.3% of the depressed single mothers had an average monthly household income of less than two million KRW, approximately half (50.8%) of the non-depressed single mothers were in this income category, indicating a significantly higher rate of low income among the depressed single mothers relative to their non-depressed counterparts (p = 0.004). At 50.8%, the majority of the depressed single mothers lived in Jeonse rental housing, and this was followed by public rental housing (at 27.7%) and monthly rental housing (18.5%); home ownership among this group of mothers was only 3.1%. Meanwhile, among the non-depressed single mothers, Jeonse rental housing was also in the majority, at 63.1%; this was followed by own homes (at 16.2%), monthly rental housing (15.4%), and public rental housing (5.4%). This indicated a higher rate of monthly rental housing and public rental housing among the depressed single mothers than among their non-depressed counterparts (p < 0.001). In the control group, no significant differences were found between the depressed and the non-depressed in terms of household income or housing type.

The mean GARS score among the depressed single mothers was significantly higher than that among their non-depressed counterparts (40.1 vs. 29.5, p < 0.001). Furthermore, in the control group, the mean GARS score of the depressed mothers was also significantly higher than that of their non-depressed counterparts (36.3 vs. 18.2, p < 0.001). Regarding the AUDIT-K score, the mean score of the depressed single mothers was significantly higher than that of their non-depressed counterparts (9.5 vs. 6, p = 0.003); however, the difference between the depressed and non-depressed married mothers in the control group was not significant.

### Factors independently influencing depression in single mothers

In the group of single mothers, the total CES-D score had a moderately positive correlation with the total GARS score (r = 0.506, p < 0.001), a mild positive correlation with the total AUDIT-K score (r = 0.251, p < 0.001), and a mild negative correlation with age (r = -0.311, p < 0.001). Meanwhile, in the control group, the total CES-D score had a moderately positive correlation with the total GARS score (r = 0.582, p < 0.001) and a mild positive correlation with the total AUDIT-K score (r = 0.195, p = 0.005) (Table 3).

**Table 3:** Correlations analysis among psychological variables in single and married mothers.

Single mothers (N = 195)			
	CES-D score	GARS score	AUDIT-K score
Age	- 0.311*	- 0.241*	- 0.226*
CES-D score	1	0.506*	0.251*
GARS score		1	0.051
Married mothers (N = 357)			
	CES-D score	GARS score	AUDIT-K score
Age score	- 0.056	0.046	0.052
CES-D score	1	0.582*	0.195*
GARS score		1	0.217*

*p value < 0.01

AUDIT-K: the Korean version of the alcohol use disorder identification test, CES-D: the center for epidemiologic studies depression scale, GARS: the global assessment of recent stress

A univariate logistic regression performed to analyze depression risk factors found the following. Single mothers who were young (OR = 0.94, 95% CI 0.90–0.98, p = 0.002), had a low monthly household income (compared to a monthly household income of two to five million KRW, OR = 0.48, 95% CI 0.25–0.92, p = 0.026), live in monthly rental housing (compared to homeowners, OR = 6.30, 95% CI 125–31.75, p = 0.026) or public rental housing (compared to homeowners, OR = 27.00, 95% CI 4.97–146.75, p < 0.001), experience a high level of stress (OR = 1.09, 95% CI 1.06–1.12, p < 0.001), and have a serious alcohol-use problem (OR = 1.07, 95% CI 1.03–1.12, p = 0.002) were more likely to become depressed. In the control group, sociodemographic variables were not correlated with depression. However, a high level of stress (OR = 1.17, 95% CI 1.12–1.22, p < 0.001) and a serious alcohol-use problem (OR = 1.11, 95% CI 1.00–1.23, p = 0.048) were also depression risk factors among this group of mothers (Table 4).

**Table 4:** Association of depression with risk factors by univariate logistic regression analyses.

	Single mothers (N = 195)				Married mothers (N = 357)			
	B	S.E,	OR (95% CI)	p	B	S.E,	OR (95% CI)	p
Age	- 0.07	0.02	0.94 (0.90–0.98)	0.002*	0.00	0.02	1.00 (0.96–1.04)	0.992
Education level								
Middle school graduate			1				1	
High school graduate	1.00	0.80	2.69 (0.57–12.66)	0.211	1.35	1.04	3.87 (0.50–29.92)	0.194
Above college	1.27	0.82	3.55 (0.71–17.72)	0.123	1.13	1.07	3.11 (0.39–25.10)	0.287
Monthly Income (10 thousand won)								
< 200			1				1	
200–499	- 0.74	0.33	0.48 (0.25–0.92)	0.026*	- 0.93	0.81	0.40 (0.08–1.93)	0.251
≥ 500	-	-	-	-	- 1.33	1.07	0.27 (0.03–2.15)	0.213
Residence								
Owner			1				1	
Jeonse rental housing	1.44	0.77	4.23 (0.94–19.05)	0.061	0.28	0.41	1.32 (0.59–2.96)	0.504
Monthly rental housing	1.84	0.83	6.30 (1.25–31.75)	0.026*	-18.69	15191.52	0	0.999
Public rental housing	3.30	0.86	27.00 (4.97–146.75)	< 0.001*	1.13	1.15	3.10 (0.33–29.2)	0.323
GARS score	0.08	0.02	1.09 (1.06–1.12)	< 0.001*	0.16	0.02	1.17 (1.12–1.22)	< 0.001*
AUDIT-K score	0.07	0.02	1.07 (1.03–1.12)	0.002*	0.10	0.05	1.11 (1.00–1.23)	0.048*

*P < 0.05

CI: Confidence interval, OR: Odds ratio, S.E.: standard error. AUDIT-K: the Korean version of the alcohol use disorder identification test, GARS: the global assessment of recent stress.

To identify the independent depression risk factors, multivariate logistic regression was performed using as covariates the variables that were correlated in the univariate logistic regression. For the single mothers, age, household income, housing type, total GARS score, and total AUDIT-K score were selected as covariates; meanwhile, for the control group, total GARS score and total AUDIT-K score were selected as covariates. Among the single mothers, living in public rental housing was an independent depression risk factor, with single mothers in such housing having 11.4 times higher odds of depression than their home-owning counterparts (OR = 11.46, 95% CI 1.72–76.46, p = 0.012). On the other hand, for the control group stress was an independent depression risk factor (OR = 1.16, 95% CI 1.09–1.24, p < 0.001) (Table 5).

**Table 5:** Multivariate logistic regression for predicting depression in single and married mothers.

		B	S.E.	OR (95% CI)	p
Single mothers (Residence)	Owner			1	
	Jeonse rental housing	1.03	0.87	2.81 (0.51–15.40)	0.233
	Monthly rental housing	0.88	0.93	2.40 (0.39–14.94)	0.347
	Public rental housing	2.44	0.97	11.46 (1.72–76.46)	0.012*
Married mothers	GARS score	0.15	0.03	1.16 (1.09–1.24)	< 0.001*

*P < 0.05

Adjusted age, monthly income, residence, GARS score, and AUDIT-K score in single mothers. Adjusted GARS and AUDIT-K score in married mothers. CI: Confidence interval, OR: Odds ratio, S.E.: standard error. AUDIT-K: the Korean version of the alcohol use disorder identification test, GARS: the global assessment of recent stress.

## Discussion

The present study examined single mothers in an attempt to identify the sociodemographic characteristics and psychological factors that contribute to depression among this population. The mean CES-D (depression scale) score for the single mothers was 20.9 points, which approximated to the cut-off score of 21 points that is typically used in epidemiologic research to determine a significant level of depression; meanwhile, the mean score among the married mothers was 12.5 points. This suggests that single mothers generally have a significantly higher depressive symptom score. On an individual basis, applying the CES-D cut-off score of 21 points showed that approximately half (97) of the 195 single mothers were depressed; however, only 12.9% (46) of the 357 married mothers had depressive symptoms. Furthermore, when the CES-D cut-off score of 25 points was applied, 33% of the single mothers and 8% of the married mothers were found to be depressed, indicating that the prevalence of depression among the single mothers was four times that of the married mothers.

The 2015 Single Parent Household Survey found that single parents have significantly poor physical and mental health, and that a great many of them suffer from depression. In fact, 20.2% responded affirmatively to the survey item, “over the previous year, I have felt sadness and despair that hampered my normal daily activities for two consecutive weeks or more,” which is almost double the 10.3% reported in the 2013 National Health Statistics survey. Furthermore, of the respondents to the former survey, the majority of single mothers reported poor subjective health status and experience of depression [2]. This shows that single mothers typically have poorer mental and physical health relative to the general population or married mothers, and it has been suggested that the risk factors that contribute to their compromised physical and mental well-being include economic hardship and weak social support [4,14,15]. In a similar vein, the 2015 Single Parent Household Survey also reported that depressive symptoms among the respondents increased as educational and income levels decreased [2].

The mean age of the single mothers was 42.7 years, which is not significantly different from the mean age of the married mothers of two-parent households. In fact, it is also similar to the 43.1 years found among the 2015 Single Parent Household Survey respondents. Furthermore, although, no significant age difference was found between the control group’s depressed and non-depressed married mothers, the mean age of the depressed single mothers was lower than that of the non-depressed single mothers. This may be because younger single mothers are more likely to have had a shorter adjustment period, and are more likely to have young children, who tend to impose greater parenting responsibilities [16]. According to the 2015 Single Parent Household Survey, single parents under the age of 30 years have a greater need for support in the areas of living costs/child-rearing costs, childcare services, and parenting education/counselling than single parents in their 40s or older; meanwhile, these former individuals also have a relatively lower need for support in the areas of housing, jobs/employment, and legal support, indicating that the need for child-rearing support is predominant among younger single parents [2].

Education level among the single mothers was relatively low, with a smaller number of college graduates present among this demographic than among the married mothers. However, for the single and married mothers alike, no significant differences in education level were found between the depressed and the non-depressed. Meanwhile, a comparison of occupations found that 66.1% of the married mothers were homemakers, and that 87% of the single mothers worked outside the home, with the majority of these latter mothers being employed in the service industry or as menial workers. However, for the single and married mothers alike, no significant occupational differences were found between the depressed and the non-depressed. Education level and occupation are linked to economic status, which can influence depressive symptoms. In other words, a person with a higher level of education attainment is more likely to have stable and reliable employment, which subsequently raises the likelihood of having a higher income and more abundant psychological and economic resources, which contribute to coping with stress. In short, such people have a lower risk of developing depression [17]. The 2015 Single Parent Household Survey also reported that single parents with a middle school degree are more likely to experience depressive symptoms than those with a high school degree or higher, and that unemployed single parents or single parents with temporary/daily employment are also more likely to experience depressive symptoms than single parents who are permanently employed, business-owning, or who work for a family business without pay [2]. Single parents with low educational attainment are more likely to receive government support as beneficiaries of national basic livelihood [2], and belonging to a socioeconomically disadvantaged group tends to increase the hardships they experience regarding raising and educating their children, which subsequently contributes to increased rates of depression [16].

In the present study, however, no significant differences in education level or employment/occupation were found between the depressed and non-depressed single mothers. As single mothers are generally forced to manage the responsibilities of both earning an income and child rearing, many of these mothers tend to seek hourly or temporary positions. For this reason, their employment options are more limited, which may explain the relatively small difference in occupational choices in this group of mothers, regardless of their education level. In fact, single mothers with a high level of educational attainment may experience a greater level of psychological duress as a result of a greater sense of relative deprivation. The fact that 90% of the single mothers in the present study had a minimum of a high school degree but held menial jobs suggests a limited range of occupational choices that is independent of educational attainment. Although being employed and able to support oneself and one’s family provides the psychological benefits of economic independence and increased self-esteem [18–20], the results of the present study illustrate the extreme economic disadvantages single mothers experience, which cannot be overcome by individuals’ efforts alone.

Previous studies have shown that the economic hardships felt by single-parent families play a significant role in mothers’ mental health [4,21] One study reported that economic deprivation increases the sense of depression in single parents, while gender and socioeconomic variables do not [22]. Moreover, another study that compared single mothers and single fathers reported that, while social discrimination and monthly income are the most significant factors influencing depressive symptoms among single mothers, self-esteem is the most important factor influencing depression among single fathers [23]. Another study also reported that, while single mothers suffer from economic deprivation, difficulties associated with child-rearing, and social prejudices, single fathers tend to suffer more from child-rearing difficulties and social prejudices than economic hardship [24], a finding that suggests that economic problems are a major factor contributing to depression among single mothers.

Over half of the single mothers analyzed in the present study had a lower average monthly household income (less than two million KRW) than the married mothers in the control group, and the depressed single mothers had a lower average monthly income than their non-depressed counterparts. Such results support the findings of previous studies in which economic hardship has been found to exert a significant influence on single mothers’ depression. The 2015 survey also found an average income of 1.74 million KRW among single-parent households, with single-parent households headed by mothers having the lowest average income, at 1.48 million KRW (such a level of income is one quarter that of the average national monthly household income); furthermore, it also reported that more economically disadvantaged single parents had depression than their more well-off counterparts [2].

In terms of housing type, home-ownership was minimal among the single mothers in the present study, indicating a less stable housing environment relative to the control group. Jeonse rental housing is a type of lease in which the landlord returns the deposit at the end of the lease term. In general, people who rent a house for Jeonse rent are in a better economic situation than those who rent on a monthly basis or public lease. Single mothers living in monthly rental housing had a 6.3 times higher risk of depression than their home-owning counterparts; furthermore, single mothers living in public rental housing had a 27 times higher risk of depression, suggesting that housing issues are a critical factor influencing depression among single mothers. The 2015 survey also found a very low home-ownership rate of 21.2% among single parents, and an even lower rate of 16.5% among single mothers. Specifically, the homeownership rate was markedly lower among single parents who were in their 30s or younger, had low educational attainment, were female, had young children not yet of school-age, were temporary workers, daily laborers, or unemployed, and who had low income [2].

Stress level was significantly higher among the single mothers than among the married mothers. For the single and married mothers alike, stress level was higher among the depressed individuals, which is consistent with previous study findings.

A higher alcohol-use score was found among the single mothers than their married counterparts. When based on the AUDIT-K cut-off of 12 points, the rate of estimated alcohol use disorders was significantly higher in single mothers (25.6%) than in the control group (8.4%). Focusing on the single mothers, a higher alcohol use score was found among the depressed than the non-depressed. Based on the AUDIT-K cut-off point, 38.5% of the depressed single mothers had suspected alcohol use disorders compared with 28.6% of the depressed married mothers. In the non-depressed group, 6.9% of the married mothers were found to have alcohol use disorders, compared with 19.2% of the single mothers. This result indicated that alcohol use problems were more prevalent among the depressed single mothers. Alcohol use in women is highly correlated with emotional factors, and it has been reported that for many women with alcohol abuse problems, a traumatic event such as marital stress, divorce, or the death of a loved one tends to be a trigger [25]. Furthermore, because many women with a drinking problem tend to have begun to use alcohol as a way of escaping from negative emotions [26], it appears that for many, depression contributes to secondary alcohol addiction [27,28]. It can be expected that single mothers are more likely to abuse alcohol to obtain relief from negative feelings such as loneliness and depression stemming from conflicts with ex-spouses, divorce, or spousal death, and that the more depressed the mother is, the more severe the alcohol-abuse problem is likely to be.

Among the single mothers, the risk of depression increased as household income level decreased, as residential instability increased, as the level of stress increased, and as the severity of drinking problems increased. Meanwhile, among the married mothers, depression risk increased with increases in stress levels. When depression risk factors in each group were controlled for, living in public rental housing was determined to be an independent depression risk factor among the single mothers, and stress was found to be an independent depression risk factor among the married mothers. In other words, for the single mothers, factors directly associated with economic stability, such as household income and housing type, were significant factors of depression, rather than emotional factors, such as stress. This finding illustrates that single mothers’ difficulties stem more from economic hardships and instability than from psychological pain. As there are limits to the actions an individual can take to overcome the economic and parenting hardships associated with single parenthood, practical social resources and policies are necessary to support these parents. In fact, the 2015 survey found that, at 65.7%, a significant share of the single parents surveyed cited “cash assistance for basic needs and child-rearing” as the most urgent need, and this was followed by those who cited “housing support such as facilities and public rental housing” (13.5%), “healthcare assistance for parents and children” (5.7%), and “childcare support services” (5.5%). These results suggest that assistance with basic needs such as stable housing and provision of childcare-related support must be prioritized when designing relevant policies. Furthermore, despite the high prevalence of depression among single parents, the economic costs and logistics associated with seeking professional care pose a great barrier. Therefore, a variety of easily accessible mental health support services must be provided to promote the mental well-being of these parents.

The limitations of the current study include the following. One, the sample size of single mothers with depression was small and generalizing the results requires caution. Furthermore, because the subjects consisted of single parents sampled from a single city, generalizing the findings across all single mothers requires caution, and the influence of the city’s geographic characteristics cannot be disregarded. At 59%, the survey response rate among single-parent households was low, which may have introduced a selection bias. To minimize these issues, both the single-parent and two-parent households in the study were selected using a stratified sampling method, which produced a more balanced selection. Furthermore, because the control group consisted of married mothers residing in the same city, the issues that the single mothers experienced could have been examined more clearly. Second, the self-report design of the questionnaire was not conducive to a more objective assessment of participants’ symptoms, and the possibility that respondents exaggerated or understated their problems cannot be disregarded. Although the CES-D, in particular, is a tool commonly used to evaluate depression in epidemiologic research, it is difficult to diagnose depression based on CES-D scores alone. However, by using a cut-off score of 25 points, which has been suggested in many previous Korean epidemiologic studies, the limitations of the self-reporting scale were minimized as much as possible. Third, because this is a cross-sectional study, the causal relationships between the variables are difficult to deduce; hence, a long-term prospective study is necessary to address this.

Despite these limitations, the current study’s contribution lies in having analyzed the risk factors of depression (the most common psychological difficulty among single mothers) through a comparison involving a control group consisting of married mothers of two-parent households. Consequently, the findings provide basic data needed to design further research and policies. The present study’s findings also suggest that practical economic assistance is urgently needed to prevent and manage depression in single mothers, and that a variety of services and programs designed to provide parenting and emotional support are necessary.

## Conclusion

Assessing depressive symptoms in single mothers and analyzing the risk factors can be helpful for designing the policies and services required to promote mental health in single-parent households. Compared with married mothers of two-parent households, major depression risk factors among single mothers relate more to economic hardship than psychological duress. Policy support such as economic assistance and child-rearing support services, in addition to other social supports, appear to be critical in preventing depression and promoting mental health among single mothers.

## References

[1] Korean Statistical Information System. Available from: http://www.index.go.kr/potal/main/EachDtlPageDetail.do?idx_cd=1578. Cited 27 June 2017

[2] Ministry of Gender Equality and Family. A study on the status of single-parent families Seoul: Ministry of Gender Equality and Family; 2015.

[3] Park SK. structural diversity of modern families and policy implications. Health and walfare policy forum. 2005;103: 73–88.

[4] Crosier T, Butterworth P, Rodgers B. Mental health problems among single and partnered mothers. The role of financial hardship and social support. Soc Psychiatry Psychiatr Epidemiol. 2007;42(1):6–13. Epub 2007 /01/03. 10.1007/s00127-006-0125-4 . 17203237

[5] Cairney J, Boyle M, Offord DR, Racine Y. Stress, social support anddepression in single and married mothers. Soc Psychiatry Psychiatr Epidemiol. 2003;38(8):442–9. 10.1007/s00127-003-0661-0 . 12910340

[6] Wang JL. The difference between single and married mothers in the 12-month prevalence of major depressive syndrome, associated factors and mental health service utilization. Soc Psychiatry Psychiatr Epidemiol. 2004;39(1):26–32. Epub 2004/03/17. 10.1007/s00127-004-0699-7 . 15022043

[7] Radloff LS. The CES-D Scale:A Self-Report Depression Scale for Research in the General Population. Appl Psychol Meas. 1977;1(3):385–401. 10.1177/014662167700100306

[8] Cho MJ, Kim KH. Diagnostic validity of the CES-D(Korean version) in the assessment of DSM-III-R major depression. J Korean Neuropsychiatr Assoc. 1993;32(3):381–99.

[9] Linn MW. A Global Assessment of Recent Stress (GARS) Scale. Int J psychiatry med. 1985;15(1):47–59. Epub 1985/01/01. . 4055247 10.2190/xp8n-rp1w-ye2b-9q7v

[10] Koh KB, Park JK. Validity and reliability of the Korean version of the Global Assessment of Recent Stress Scale. Korean Journal of Psychosomatic Medicine. 2000;8(2):201–11.

[11] Saunders JB, Aasland OG, Babor TF, de la Fuente JR, Grant M. Development of the Alcohol Use Disorders Identification Test (AUDIT): WHO Collaborative Project on Early Detection of Persons with Harmful Alcohol Consumption—II. Addiction. 1993;88(6):791–804. Epub 1993/06/01. . 8329970 10.1111/j.1360-0443.1993.tb02093.x

[12] Babor TF, Higgins-Biddle JC, Sauders JB, Monteiro MG. The Alcohol Use Disorders Identification Test, 2nd ed World Heath Organization; 2001 Available from: http://apps.who.int/iris/bitstream/handle/10665/67205/WHO_MSD_MSB_01.6a.pdf. Cited 01 Aug 2018.

[13] Lee BO, Lee CH, Lee PG, Choi MJ, Namkoong K. Development of Korean Version of Alcohol Use disordiers Identification Test(AUDIT-K): Its Reliability and Validity. J Korean Academy of Addiction Psychiatry 2000;4(2):83–92.

[14] Targosz S, Bebbington P, Lewis G, Brugha T, Jenkins R, Farrell M, et al Lone mothers, social exclusion and depression. Psychological medicine. 2003;33(4):715–22. Epub 2003/06/06. . 12785473 10.1017/s0033291703007347

[15] Benzeval M. The self-reported health status of lone parents. Soc Sci Med. 1998;46(10):1337–53. Epub 1998/07/17. . 9665565 10.1016/s0277-9536(97)10083-1

[16] Byun WS, Song DY, Kim YR. A study on the living conditions and welfare needs of the family type Seoul: Korean Women’s Development Institute; 2001.

[17] Bjelland I, Krokstad S, Mykletun A, Dahl AA, Tell GS, Tambs K. Does a higher educational level protect against anxiety and depression? The HUNT study. Soc sci med. 2008;66(6):1334–45. Epub 2008/02/01. 10.1016/j.socscimed.2007.12.019 . 18234406

[18] Bambra C. Yesterday once more? Unemployment and health in the 21st century. J Epidemiol Community Health. 2010;64(3):213–5. 10.1136/jech.2009.090621 20203122

[19] Bartley M. Unemployment and ill health: understanding the relationship. J Epidemiol Community Health. 1994;48(4):333–7. ; PMCID: PMC1059979. 7964329 10.1136/jech.48.4.333 PMC1059979

[20] Ok SW, Choi SE, Kwon K, Kang EG. The Social Support Network of Divorced Single Mother Families. Journal of Korean Home Management Association. 2004;22:181–91.

[21] Kim DS, Jeon GS, Jang SN. Socioeconomic status, social support and self-rated health among lone mothers in South Korea. Int J Public Health. 2010;55(6):551–9. Epub 2010/07/09. 10.1007/s00038-010-0169-9 . 20614228

[22] Kim MS, Won YH. The Impact of self-control on the low-income single parents' subjective well-being. The Women. 2006;71:75–105.

[23] Kim JR, Kim HS. The effect of variables on depression of single parents family householder. Journal of Family Relations. 2014;19(1):143–60.

[24] Hwang ES. Comparison of conflicts in single mother families and single father families. J Kor Single Parent Fam Institute. 2007;6:1–20.

[25] Kim HR, Choi YH, Choi JK. A Study on the Characteristics of Women Problem Drinker and Their Environment. J Korean Alcohol Sci. 2003;4(2):105–18.

[26] Lim SY, Cho HS, Lee YH. A case study about female alcoholic's addictive process. Kor J Clin Psychol. 2005;24(4):869–86.

[27] Helzer JE, Pryzbeck TR. The co-occurrence of alcoholism with other psychiatric disorders in the general population and its impact on treatment. J Stud Alcohol. 1988;49(3):219–24. Epub 1988/05/01. . 3374135 10.15288/jsa.1988.49.219

[28] Dixit AR, Crum RM. Prospective study of depression and the risk of heavy alcohol use in women. Am J psychiatry. 2000;157(5):751–8. Epub 2000/04/28. 10.1176/appi.ajp.157.5.751 . 10784468

